# Transcriptome-wide analysis of a baculovirus using nanopore sequencing

**DOI:** 10.1038/sdata.2018.276

**Published:** 2018-12-04

**Authors:** Zsolt Boldogkői, Norbert Moldován, Attila Szűcs, Dóra Tombácz

**Affiliations:** 1 Department of Medical Biology, Faculty of Medicine, University of Szeged, Szeged, 6720, Hungary

**Keywords:** RNA sequencing, Transcriptomics, Virology

## Abstract

*Autographa californica multiple nucleopolyhedrovirus* (AcMNPV) is a prototypic baculovirus infecting specific insects. AcMNPV contains a large double-stranded DNA genome encoding a complex transcriptome. This virus has a widespread application as a vector for the expression of heterologous proteins. Here, we present a dataset, derived from Oxford Nanopore Technologies (ONT) long-read sequencing platform. We used both cDNA and direct RNA sequencing techniques. The dataset contains 520,310 AcMNPV and 1,309,481 host cell reads using the regular cDNA-sequencing method of ONT technique, whereas altogether 6,456 reads were produced by using direct RNA-sequencing. We also used a Cap-selection protocol for certain ONT samples, and obtained 2,568,669 reads by using this method. The raw reads were aligned to the AcMNPV reference genome (KM667940.1). Here, we openly released the ‘static’ and the dynamic transcript catalogue of AcMNPV. This dataset can be used for deep analyses of the transcriptomic and epitranscriptomic patterns of the AcMNPV and the host cell. The data can be also useful for the validation of different bioinformatics software packages and analysis tools.

## Background & Summary

Baculoviruses are a diverse group of viruses infecting insect larvae of the orders *Diptera, Hymenoptera, and Lepidoptera*^1^. By far the most widely studied member of *Baculoviridae* family is the *Autographa californica* nucleopolyhedrovirus (AcMNPV), of which the complete genome sequence has been determined and annotated^2^. AcMNPV has a circular, double-stranded 134-kpb DNA genome, packaged in a rod-shaped capsid. AcMNPV has been used as a model in studies of the molecular pathogenesis of baculoviruses^3^. Additionally, this virus is a popular vector for the expression of heterologous proteins^4^.

Although the previously published short-read Illumina RNA sequencing provides high-quality data^5^, the long-read sequencing (LRS) dataset is more beneficial; the LRS methods are more applicable for global RNA profiling, as they can greatly improve and expand the reference set of transcripts^6–14^, even if they have a relatively high error-rate and a low throughput.

In this study, the Oxford Nanopore Technologies (ONT) MinION long-read sequencing device was used to characterize the static and dynamic (including nine different post-infection time points) AcMNPV and host cell (*Spodoptera frugiperda* isolate Sf9) transcriptomes following various library preparation approaches, such as full-length cDNA-sequencing, direct RNA-sequencing (dRNA-seq) to avoid the potential false products from reverse-transcription or PCR, and cDNA-sequencing on 5′ Cap-selected samples (Cap-seq) for the more precise detection of the transcription start sites. The applied full-length sequencing protocols capture the entire polyA(+) fraction of AcMNPV transcriptome.

Two MinION flow cells were used for the cDNA sequencing, while one and two flow cells were applied for the Cap-seq and dRNA-seq methods, respectively.

**Figure 1:**
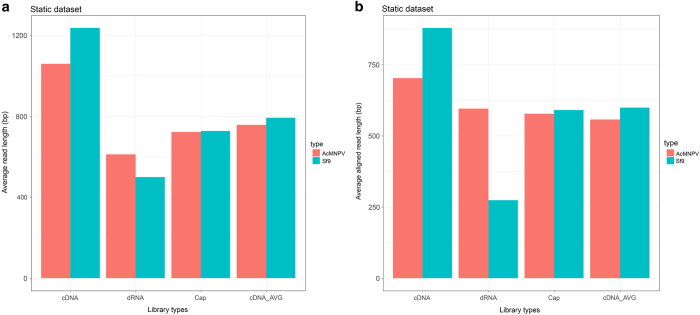
Barplot figure shows the read lengths of the static dataset. (**a**) The figure illustrates the average read lengths of the cDNA-Seq, dRNA-Seq and Cap-Seq samples, as well as the weighted arithmetic mean values from the individual time points. (**b**) This plot shows the average mapped read lengths of the different samples. AVG: Weighted arithmetic mean.

**Figure 2:**
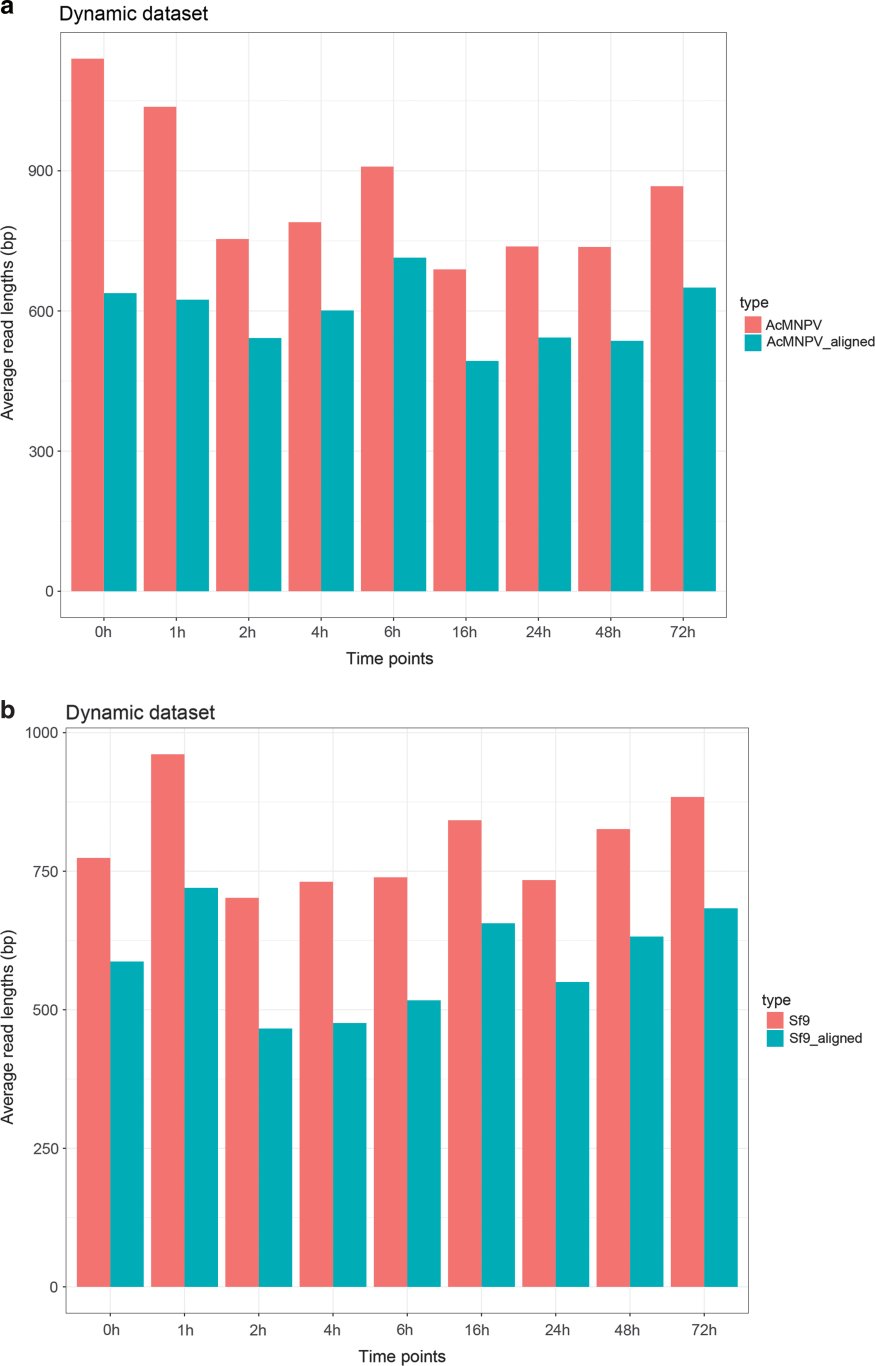
Barchart diagram represents the sequencing read length of the cDNA-sequencing of samples from various time points. (**a**) AcMNPV (**b**) Sf9.

The cDNA sequencing of the static (mixed) sample yielded altogether 95,953 sequencing reads mapped to the AcMNPV genome strain E2 (GeneBank accession: KM667940.1) with an average coverage of 503x; (Table 1). Table 2 contains the detailed information about the read counts from different time points. The Cap-seq method resulted in 488,847 reads (2,108-fold coverage) AcMNPV-specific reads. The average read-lengths for the cDNA-seq vary between 689–1139 bp, while the Cap-seq and dRNA-seq resulted in an average read length of 718 bp and 613 bp, respectively (Table 2, Figs 1 and 2). The reads were also aligned to the host cell genome (BioProject accession: PRJNA380964, Tables 3 and 4, Figs 1 and 2).

Here, we present a large dataset of AcMNPV transcriptome derived from LRS experiments for the first time, including various techniques applied (Fig. 1). Our aim with this study was to provide a dataset that can be used for identifying mRNAs, non-coding transcripts, and transcript isoforms, such as the transcriptional start and end sites, along with splice variants of this baculovirus, as well as to define full-length transcripts by using a combination of various library preparation approaches for long-read sequencing. The barcoded samples derived from different time points can also be used for the kinetic characterization of AcMNPV transcripts, as well as for the analysis of gene expression of the host cells during viral infection^15^. Moreover, these data allow the comparison of different library preparation methods. Furthermore, our dataset can also be used for the identification of modified nucleotides and for obtaining epitranscriptomic data.

## Methods

**Figure 3:**
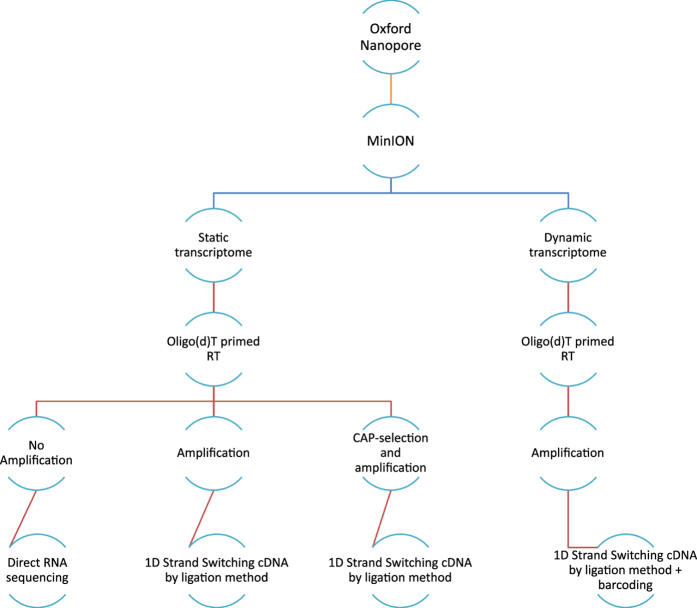
Data flow diagram shows the detailed overview of the study design.

Figure 3 gives an overview of the methodological workflow used in the present study. The utilized reagents are listed in Table 5.

### Cells, viruses and infection conditions

The Sf9 epithelial cell line (derived from the parental *Spodoptera frugiperda* cell line) was used for the propagation of a LacZ expressing recombinant *Autographa californica multiple nucleopolyhedrovirus* (AcMNPV) in Sf-900 II SFM insect cell culture medium (Thermo Fisher Scientific). Cultivation of the infected cells was carried out in Corning Spinner Flasks (Sigma Aldrich/Merck) at 26 °C, the speed of the shaker was set to 70 rpm. The LacZ gene was inserted to the promoter region of *polh* gene (βgal-AcMNPV). The virus stock was obtained from SOLVO Biotechnology Inc. (Szeged, Hungary). Cells were infected with a multiplicity of infection of 2 plaque forming unit/cell. After infection, cells were incubated for 0, 1, 2, 4, 6, 16, 24, 48 or 72 h pi, then five ml from the samples were centrifuged at 2000 rpm at 4°C, followed by washing with phosphate-buffered saline (PBS) and centrifuged again by setting the same parameters. Cells were stored at −80°C until use.

### RNA extraction

#### Purification of total RNA

The NucleoSpin^®^ RNA kit (Macherey-Nagel) was used to isolate RNA from viral infected cells for sequencing, as was described in our previous publications^10^. In short, samples were lysed by incubation in a chaotropic ion containing solution (supplied by the kit), then samples were treated with DNase I solution (provided by the kit). Total RNAs were eluted from the membrane in RNase-free water. To remove the probable residual DNA contamination, samples were handled by Ambion® TURBO DNA-free™ Kit (Thermo Fisher Scientific). The RNA samples were quantified by Qubit®. 2.0 Fluorometer using Qubit RNA BR Assay Kit (Life Technologies) and then they were stored at −80 °C until further use.

#### Ribosomal RNA depletion

For the CAP-selection protocol, the ribosomal RNA (rRNA) was eliminated from the total RNA samples using the Epicentre Ribo-Zero™ Magnetic Kit H/M/R (Illumina).

#### Isolation of polyadenylated RNA

For the cDNA and Direct RNA sequencing, the polyA(+) fraction of the RNA samples were purified using the Qiagen Oligotex mRNA Mini Kit, according to the “Spin Columns” protocol of the kit.

The final concentrations of the rRNA depleted and the PolyA(+) RNA samples were determined with Qubit RNA HS Assay Kit (Life Technologies).

### cDNA synthesis, library preparation and sequencing

The Oxford Nanopore Technologies MinION real-time device was used for cDNA and direct RNA sequencing.

### Oxford Nanopore 1D cDNA sequencing - mixed RNA sample

Viral and Sf9 transcripts were sequenced on MinION sequencer following the 1D Strand switching cDNA by ligation protocol (Version: SSE_9011_v108_revS_18Oct2016). The ONT Ligation Sequencing Kit 1D (SQK-LSK108) was used for the library preparation. The PolyA(+)-selected RNAs were used for cDNA production. Equal amount of RNAs from the different time points were mixed together.

75 ng from the mixed sample (Table 6) was subjected to reverse transcription. Poly(T)-containing anchored oligonucleotides [(VN)T_20_; ordered from Bio Basic, Canada, (Table 7)] and dNTPs (10 mM, Thermo Fisher Scientific) was added to the RNA. The sample was incubated at 65°C for 5 min and then the strand-switching oligo [containing three O-methyl-guanine RNA bases (PCR_Sw_mod_3G; Bio Basic, Canada)], buffer and DTT [both are supplemented by the SuperScript IV Reverse Transcriptase kit (Life Technologies)] were added. The mixture was treated with a recombinant ribonuclease inhibitor (RNase OUT™, Life Technologies), and then it was incubated for 2 min at 42 °C. Two-hundred units from the 200U SuperScript IV Reverse Transcriptase enzyme were added to the sample. The generation of the first strand cDNA was performed at 50 °C for 10 min, then the strand switching step [when the reverse transcriptase adds 1–3 non-templated cytosines to the 3’ end of the cDNA strand, and a primer (which was added to the RT reaction) anneals to the non-templated cytosines. This incorporates a PCR-priming sequence to the end of the full-length cDNAs] at 42 °C for 10 min. The enzyme inactivation step was at 80 °C for 10 min. Five μl from the cDNA was amplified using KAPA HiFi DNA Polymerase enzyme (Kapa Biosystems) and Ligation Sequencing Kit Primer Mix (included in the 1D Kit). The Applied Biosystems Veriti Thermal Cycler was used following the ONT 1D Kit’s recommendations: preliminary denaturation at 95 °C for 30 s (1 cycle); denaturation for 15 sec at 95 °C (15 cycles); annealing for 15 sec at 62 °C (15 cycles); extension for 4 min at 65 °C (15 cycles); final elongation step was for 10 min at 65 °C. NEBNext End repair/dA-tailing Module (New England Biolabs) was applied for end repair, whereas NEB Blunt/TA Ligase Master Mix (New England Biolabs) was utilized for adapter ligations. The adapter sequences were provided by the 1D kit. Agencourt AMPure XP magnetic beads (Beckman Coulter) were used for sample purification after each enzymatic reaction. For the quantification of the libraries, the Qubit Fluorometer 2.0 and the Qubit (ds)DNA HS Assay Kit (both from Life Technologies) was used. The sequencing-ready libraries were loaded on R9.4 SpotON Flow Cells, and the Albacore v1.2.6 software was used for base calling.

### Oxford Nanopore 1D cDNA sequencing – different time points

For the analysis of the dynamic changes of the global full-length transcriptome of the AcMNPV, the RNA samples from different post infection time-points were individually sequenced. CDNAs were generated from the polyA(+) RNA samples from 0, 1, 2, 4, 6, 16, 24, 48 or 72 h pi. The starting amounts of RNA samples are summarized in Table 7. The preparation of cDNA libraries was carried out according the above mentioned 1D protocol, until the end-repair step. After this, the 1D PCR barcoding (96) genomic DNA protocol (version: PBGE96_9015_v108_revS_18Oct2016, updated 25/10/2017) was followed, starting with the Barcode Adapter ligation step. Ten μl from the samples were mixed with 6.5 μl Barcode Adapter (Table 8) and 17 μl Blunt/TA Ligase Master Mix (1/3 reaction volumes were applied compared to the protocol’s recommendations). After 10 min incubation at room temperature, the samples were purified by using XP beads. Samples were amplified by PCR using KAPA HiFi DNA Polymerase and 1 μl from one of the PCR Barcodes (Table 8), as recommended by the 1D PCR barcoding protocol. Table 8 shows the sample concentrations after the amplification. Samples were mixed together according to the Table 8 and altogether two libraries were prepared from them. The second end-prep and adapter ligation steps were carried out following the previously mentioned protocol. The ready libraries were washed by XP beads, and then they were load on the SpotON flow cell.

#### MinION cDNA sequencing on Cap-selected samples

The TeloPrime Full-Length cDNA Amplification Kit (Lexogen) was used to obtain full-length, capped RNAs with the exact 5’-ends. A mixed total RNA sample (containing RNA from 1, 2, 4, 6, 16, 24, 48 and 72 h pi) was subjected to rRNA-depletion and then, 216 ng from the ribo-depleted sample was used for reverse transcription (RT), double-stranded (ds)cDNA production and library preparation. First, the RNA was mixed with RT buffer and an oligodT containing primer (both are derived from the kit, Table 7). The RT mixture was heated to 70 °C for 30 seconds and then it was cooled down to 37 °C for 1 min. The RT enzyme and reagents (supplied by the kit) were added to the sample and the reaction was kept at 37 °C for an additional 2 min. Temperature was increased to 46 °C for 50 min. Silica columns (from the Lexogen kit) were used to purify the RNA-cDNA hybrid. An adapter was ligated to the cDNA by base-pairing of the 5’C to the cap structure of the RNA templates by using a double-strand specific ligase from the kit. Ligation reaction was carried out at 25°C, overnight, then the sample was purified using the silica columns. The Second-Strand Mix and the Enzyme Mix from the Teloprime kit were used to produce dscDNA. The cDNA synthesis was performed in a Veriti PCR thermal cycler, applying the following settings: 98 °C for 30 s, 50 °C for 90 s, 72 °C for 5 min, 35 cycles of 98 °C for 30 s, 62 °C for 60 s, 72 °C for 5 min, and hold at 72 °C for 5 min. The Qubit dsDNA HS quantitation assay (Life Technologies) was used to measure the sample quantity. The specificity of the gained PCR product was analysed by using real-time PCR reaction: the Rotor-Gene Q qPCR cycler (Qiagen) was used, a gene specific primer (*104.1*, 10 μM each; Table 9), ABsolute qPCR SYBR Green Mix (Thermo Fisher Scientific), and cDNA was mixed in 20 μl final volume. The initial denaturation step was 94 °C for 15 min, and it was followed by 35 cycles of 94 °C for 25 s, 60 °C 25 s and 72 °C 6 s.

The PolyA(+)-CAP-selected samples were also subjected to MinION sequencing following the 1D Strand switching cDNA by ligation method. These cDNA samples were end-repaired and then they were ligated with the 1D adapters. Finally, they were loaded on the ONT R9.4 SpotON Flow Cells.

#### Direct RNA sequencing

The ONT’s Direct RNA sequencing (DRS) protocol (Version: DRS_9026_v1_revM_15Dec2016) was used for amplification-free sequencing. Total RNAs from 7 time points (1, 2, 4, 6, 16, 24, 48 and 72 h pi) were mixed together, and then the PolyA(+) fraction was isolated from the mixture. One hundred ng from the sample (Table 6) was mixed with the oligodT-containing T_10_ adapter (RT adapter; part of the ONT Direct RNA Sequencing Kit; SQK-RNA001; ONT) and T4 DNA ligase (2 M U/ml; New England BioLabs). After 10 min incubation at room temperature, the first-strand cDNA synthesis was carried out in 40 μl final volume using the SuperScript III Reverse Transcriptase enzyme (Life Technologies), following the DRS protocol: first the sample was incubated at 50 °C for 50 min, then the temperature was increased – in order to inactivate the enzyme – to 70 °C for 10 min in a Veriti PCR Cycler. Samples were purified with Agencourt AMPure XP Beads (Beckman Coulter). The beads were handled with RNase OUT (40 U/μl; Life Technologies) before usage: 2U enzyme was measured to 1 μl bead. Washed RNA-cDNA hybrids were eluted in 20 μl Ambion Nuclease-Free Water (Thermo Fisher Scientific). The sample was ligated to RMX sequencing adapter by using T4 DNA ligase and NEBNext Quick Ligation Reaction Buffer (New England BiceoLabs). Ligation reaction was performed at room temperature for 10 min. Samples were purified with the RNase inhibitor-treated XP beads using Wash Buffer (provided by the DRS Kit) and then eluted in 21 μl Elution Buffer (DRS Kit). The concentration of the adapter-ligated RNA-cDNA hybrids was detected by using the Qubit 2.0 Fluorometer and Qubit dsDNA HS Assay Kit (Life Technologies). Samples were loaded onto the R9.4 SpotON Flow Cell.

Data on the quality of sequencing reads - mapped to the AcMNPV and Sf9 genomes - including insertions, deletions, and mismatches, as well as the coverages are summarized in Tables 1–4.

### Read processing

The Albacore software v1.2.6 (ONT) was used for base calling of the data from ‘static’ cDNA and dRNA sequencing, while the newer version of the software (v.2. 0.1) was utilized for the CAP-selected and dynamic datasets. The software was able to identify barcodes on 82% of the barcoded reads. The sequencing reads were aligned by using GMAP^16^, with the following settings: gmap -d Genome.fa –nofails -f samse File.fastq > Mapped_file.sam. The Porechop tool v0.2.3 was used with default setting except using flag –untrimmed to split original fastq file by barcodes. The quality information of the dataset was obtained by using our in-house scripts.

### Code Availability

1. Albacore v2.0.1: https://github.com/Albacore/albacore

2. GMAP: http://research-pub.gene.com/gmap/ (version 2015-12-31)

3. Porechop v.0.2.3: https://github.com/rrwick/Porechop

4. Custom routines have been archived on Github (https://zenodo.org/record/1034511).

## Data Records

Data from the mixed samples including cDNA-, dRNA- and Cap-seq (Data Citation 1), as well as the data from the dynamic dataset (Data Citation 2) have been uploaded to the European Nucleotide Archive. All sequencing reads were mapped to the KM667940.1 genome. All data can be used without restrictions.

## Technical Validation

The quantity of the purified RNAs (including the total RNAs, the polyA-selected RNAs, as well as the rRNA-depleted samples), the produced cDNA samples and the final sequencing libraries were measured by Qubit 2.0 (Life Technologies) fluorometer using the Qubit RNA Broad-Range, High Sensitivity RNA and High Sensitivity dsDNA Assay Kits.

## Usage Notes

This dataset provided here, was produced primarily to determine the complexity, to analyse the isoforms (potential splice variants, as well as transcriptional start and stop site variations) and the dynamic properties of AcMNPV transcriptome. The uploaded binary alignment (BAM) files contain reads already mapped to the KM667940.1 AcMNPV reference genome, as well as to the host genome (PRJNA380964) using GMAP v2017-04-24^15^. The raw FastQ files have also been uploaded for each sample to extend the potential usage of the data.

The provided dataset can be further analysed using bioinformatics tools, such as Tombo^17^, bedtools^18^ and samtools^19^, or using different visualization tools such as the IGV^20^, Geneious^21^ or Artemis^22^. The uploaded files have not been trimmed, they contain terminal poly(A) sequences as well as the 5′ and 3′ adapters, which can be used to determine the orientations of the reads.

## Additional information

**How to cite this article**: Boldogkői, Z. *et al*. Transcriptome-wide analysis of a baculovirus using nanopore sequencing. *Sci. Data*. 5:180276 doi: 10.1038/sdata.2018.276 (2018).

**Publisher’s note**: Springer Nature remains neutral with regard to jurisdictional claims in published maps and institutional affiliations.

## Supplementary Material



## Figures and Tables

**Table 1:** Summary table of the cDNA-, dRNA and Cap-sequencing reads - from the mixed samples - mapped to the AcMNPV genome.

Sample	Number of aligned reads	Median of read lengths	Average of read lengths±SE	Average of aligned read lengths±SE	Average Insertion frequency (%)±SE (%)	Average Deletion frequency (%)±SE (%)	Average Mismatch frequency (%)±SE (%)	Coverage	N50
**cDNA-seq**	95953	840	1060.76±2.39	702.89±1.56	2.83±0.01	5.92±0.01	7.31±0.01	503.45	816
**dRNA-seq**	2425	504	612.53±8.04	595.96±8.35	2.02±0.04	8.62±0.05	6.10±0.04	10.79	678
**Cap-seq**	488847	627	723.10±0.53	577.88±0.45	2.75±0.00	3.70±0.00	4.81±0.00	2108.69	529
Read N50 is defined as the length N for which 50% of all bases in the reads are in a sequence of length L < N.									

**Table 2:** Summary statistics of the cDNA sequencing reads – derived from the different time points - aligned to the AcMNPV genome.

Sample	Number of aligned reads	Median of read lengths	Average of read lengths±SE	Average of aligned read lengths±SE	Average Insertion frequency (%)±SE (%)	Average Deletion frequency (%)±SE (%)	Average Mismatch frequency (%)±SE (%)	Coverage	N50
**0h**	135	1010	1139.68±53.61	638.67±40.74	4.05±0.21	3.51±0.17	5.84±0.25	0.64	661
**1h**	90	902	1037.20±70.43	624.51±51.52	4.08±0.29	3.52±0.22	5.72±0.26	0.42	660
**2h**	870	577	753.84±15.58	541.90±13.17	3.78±0.07	3.29±0.07	5.08±0.11	3.52	596
**4h**	21557	592	789.73±3.07	601.71±2.99	3.66±0.01	3.51±0.01	5.14±0.02	96.82	881
**6h**	19989	730	908.66±3.72	714.37±3.61	3.66±0.01	3.41±0.01	5.11±0.02	106.59	1025
**16h**	84201	582	689.09±1.27	492.74±1.23	3.63±0.01	3.17±0.01	5.14±0.01	309.69	509
**24h**	145127	593	738.07±1.04	543.22±1.01	3.66±0.01	3.14±0.00	5.11±0.01	588.47	598
**48h**	92564	617	737.39±1.26	535.93±1.14	3.68±0.01	3.16±0.01	5.15±0.01	370.30	517
**72h**	59824	633	867.47±2.41	650.31±2.16	3.56±0.01	2.99±0.01	4.94±0.01	290.40	894
Read N50 is defined as the length N for which 50% of all bases in the reads are in a sequence of length L < N.									

**Table 3:** Summary table of the cDNA-, dRNA and Cap-sequencing reads - from the mixed samples - mapped to the host genome

Sample	Number of aligned reads	Median of read lengths	Average of read lengths±SE	Average of aligned read lengths±SE	Average Insertion frequency (%)±SE (%)	Average Deletion frequency (%)±SE (%)	Average Mismatch frequency (%)±SE (%)	Coverage	N50
**cDNA-seq**	210987	942	1236.91 ±2.07	879.06±1,71	3.49±0.01	5.75±0.00	6.66±0.01	0.361	1073
**dRNA-seq**	4031	284	499.18±16.11	274.47±6.03	3.21±0.11	5.03±0.09	3.72±0.06	0.002	466
**Cap-seq**	2079822	672	726.81±0.27	591.93±0.24	2.89±0.00	4.27±0.00	5.41±0.00	2.394	617
Read N50 is defined as the length N for which 50% of all bases in the reads are in a sequence of length L<N.									

**Table 4:** Summary statistics of the cDNA sequencing reads – derived from the different time points - aligned to the Sf9 genome

Sample	Number of aligned reads	Median of read lengths	Average of read lengths±SE	Average of aligned read lengths±SE	Average Insertion frequency (%)±SE (%)	Average Deletion frequency (%)±SE (%)	Average Mismatch frequency (%)±SE (%)	Coverage	H50
**0h**	169794	680	773.75±0.99	586.81±0.94	3.69±0.01	3.77±0.01	4.94±0.01	0.19	649
**1h**	20346	642	961.12±6.74	719.88±6.50	3.54±0.02	3.24±0.02	4.54±0.02	0.03	796
**2h**	27209	588	701.67±2.77	466.31±2.64	3.41±0.02	3.53±0.02	4.62±0.02	0.02	765
**4h**	46519	611	730.90±1.90	475.63±1.87	3.36±0.02	3.35±0.01	4.44±0.01	0.04	611
**6h**	60076	627	738.99±1.68	517.17±1.59	3.52±0.01	3.58±0.01	4.72±0.01	0.06	438
**16h**	275282	683	841.67±1.11	656.40±1.08	3.85±0.00	3.54±0.00	4.92±0.00	0.35	617
**24h**	299940	641	733.99±0.71	550.25±0.69	3.79±0.00	3.61±0.00	4.96±0.00	0.32	621
**48h**	80781	687	826.03±1.86	632.02±1.77	3.83±0.01	3.58±0.01	5.00±0.01	0.10	610
**72h**	118547	750	883.51±1.32	683.21±1.28	3.97±0.01	3.79±0.01	5.19±0.01	0.16	1629
Read N50 is defined as the length N for which 50% of all bases in the reads are in a sequence of length L&lt;N.									

**Table 5:** Summary table of the reagents and chemistries used for the sequencing.

Total RNA isolation	PolyA selection	Ribodepletion	Reverse transcription & dscDNA production	cDNA synthesis by PCR	Library preparation kit
Macherey-Nagel RNA	Qiagen Oligotex mRNA mini Kit	-	SuperScript III	-	Direct RNA Sequencing Kit
			SuperScript IV	KAPA HiFi PCR Kit	Ligation Sequencing Kit 1D
					PCR Barcoding Expansion 1-96+Ligation Sequencing Kit 1D
	-	Epicentre Ribo-Zero™ Magnetic Kit H/M/R	Lexogen Teloprime Kit enzymes & reagents	Lexogen Teloprime PCR mix	Ligation Sequencing Kit 1D

**Table 6:** Overview table of the amount of utilized nucleic acids for cDNA, dRNA and Cap-seq from mixed samples.

Sample	Starting material (ng)	Amount of the library after PCR (ng)	Amount of the loaded library onto the flow cell (ng)
**cDNA**	75	475	170
**dRNA**	100	no PCR	46
**Cap-seq**	216	120	90

**Table 7:** The list of different, oligod(T)-containing primers used in this study for the reverse transcription reactions.

Sequencing method	Name, availability	Catalog #	Sequence (5′ -> 3′)
cDNA-seq	Poly(T)-containing anchored primer [(VN)T20 - ONT recommended, custom made (Bio Basic)	-	5phos/ ACTTGCCTGTCGCTCTATCTTC(T)_20_VN
dRNA-seq	RT adapter - Direct RNA Sequencing Kit (Oxford Nanopore Technologies)	SQK-RNA001	GAGGCGAGCGGTCAATTTTCCTAAGAGCAAGAAGAAGCCTTTTTTTTTT
Cap-seq	TeloPrime Full-Length cDNA Amplification Kit (Lexogen)	013.08 & 013.24	TCTCAGGCGTTTTTTTTTTTTTTTTTT

**Table 8:** Overview table of the amount of utilized nucleic acids for cDNA- seq for dynamic transcriptome analysis.

Sample	Starting material (ng)	Amount of the library after PCR (ng)	Amount of the loaded library onto the flow cell	Barcode #
**1h**	58	1224 ng	440 ng	C2
**2h**	59	684 ng		C3
**4h**	54	612 ng		C4
**6h**	53	744 ng		C5
**16h**	54.5	738 ng	410 ng	C6
**24h**	60	570 ng		C7
**48h**	52.5	351 ng		C8
**72h**	56	360 ng		C9
**0h**	50	600 ng		C1

**Table 9:** The sequence of the gene-specific primers used for the PCR amplification of 104.1 gene of AcMNPV.

Primer	5′ -> 3′
fw	AACGTGCTGTTGAATTATGTGG
rev	AAACTGTTATCAATTAGTTTCGTTT
